# Case Report: Multifocal primary cutaneous anaplastic large cell lymphoma with angioinvasive features in a child

**DOI:** 10.3389/fmed.2026.1981047

**Published:** 2026-10-29

**Authors:** Shiwen Guo, Yao Xie, Lin Wang

**Affiliations:** Department of Dermatology, West China Hospital, Sichuan University, Chengdu, Sichuan, China

**Keywords:** angiodestruction, angioinvasion, case report, CD30+ lymphoproliferative disorder, primary cutaneous anaplastic large cell lymphoma

## Abstract

Primary cutaneous anaplastic large cell lymphoma (C-ALCL) is the second most common cutaneous T-cell lymphoma after mycosis fungoides. It is rare in children and typically manifests as solitary or localized reddish-brown nodules or tumors. Histologically, it is characterized by a diffuse dermal infiltrate of medium-to-large atypical anaplastic cells. Despite the frequently alarming clinical and histologic presentation, the disease pursues an indolent course with a favorable prognosis. Herein, we report a case of C-ALCL in an 8-year-old boy presenting with multiple ulcers on the extremities, with the largest measuring 4 cm × 4 cm. Histopathological examination revealed an angiocentric and angiodestructive infiltrate composed of large atypical lymphoid cells. To our knowledge, this distinctive histopathological pattern has not previously been reported in pediatric C-ALCL and represents an important diagnostic pitfall because of its close resemblance to type E lymphomatoid papulosis and extranodal NK/T-cell lymphoma. The boy underwent surgical excision with no recurrence observed during the 10-month follow-up.

## Introduction

Primary cutaneous anaplastic large cell lymphoma (C-ALCL) is a CD30+ lymphoproliferative disorder that typically occurs in elderly patients, shows a slight male predominance, and is rarely encountered in children (1). C-ALCL typically manifests as a solitary and localized papule or nodule that may ulcerate, characterized by variable histopathological presentations and a broad cytomorphologic spectrum (2). Notably, an angiocentric/angiodestructive variant has been reported in a subset of C-ALCL cases (3). Angioinvasion/angiodestruction is a histopathological feature commonly observed in lymphomatoid papulosis type E, which, together with C-ALCL, belongs to the spectrum of CD30+ lymphoproliferative disorders. This feature is also encountered in aggressive lymphomas such as extranodal NK/T-cell lymphoma, primary cutaneous *γ*/*δ* T-cell lymphoma, and hydroa vacciniforme-like lymphoproliferative disorder, usually associated with aggressive clinical behavior and unfavorable prognosis (4). We report a pediatric case of multifocal C-ALCL presenting with rapidly enlarging and ulcerated nodules and masses on the extremities. Histopathologic examination revealed infiltration and destruction of vessel walls by atypical lymphoid cells. To date, no pediatric case of the C-ALCL variant with angiocentric/angiodestructive histopathological features has been reported. Early and accurate diagnosis, particularly differentiation from aggressive lymphomas, is essential to avoid overtreatment and ensure appropriate prognostic assessment.

## Case report

An 8-year-old boy presented with a one-month history of rapidly enlarging nodules and ulcers on the extremities. One month before presentation, following exposure to ostriches and river water, the boy developed three soybean-sized erythematous nodules on the left upper arm and bilateral knees. The central portions of the nodules gradually ulcerated, forming circular ulcers covered with thick black eschars, accompanied by mild itching and pain. The lesions progressively enlarged without spontaneous regression. The patient initially presented to an outside hospital, where the diagnosis was uncertain and topical mupirocin and hydrocortisone acetate were prescribed. As the lesions continued to enlarge, he subsequently presented to our institution, where a cutaneous infection was suspected and amoxicillin, itraconazole, and prednisone 20 mg/day were administered, without improvement. The clinical course and treatment timeline are summarized in Figure 1. He had no systemic symptoms, including fever, night sweats, arthralgia, or weight loss. He had no significant family history of lymphoma or immunodeficiency, and his growth and development were age-appropriate. Physical examination revealed no cervical, axillary, or inguinal lymphadenopathy. Three circular ulcers measuring 4 cm, 0.5 cm, and 3 cm in diameter were observed on the left upper arm, left knee, and right knee, respectively. Each ulcer had a raised erythematous border and was covered with a thick black eschar (Figure 2).

**Figure 1:**
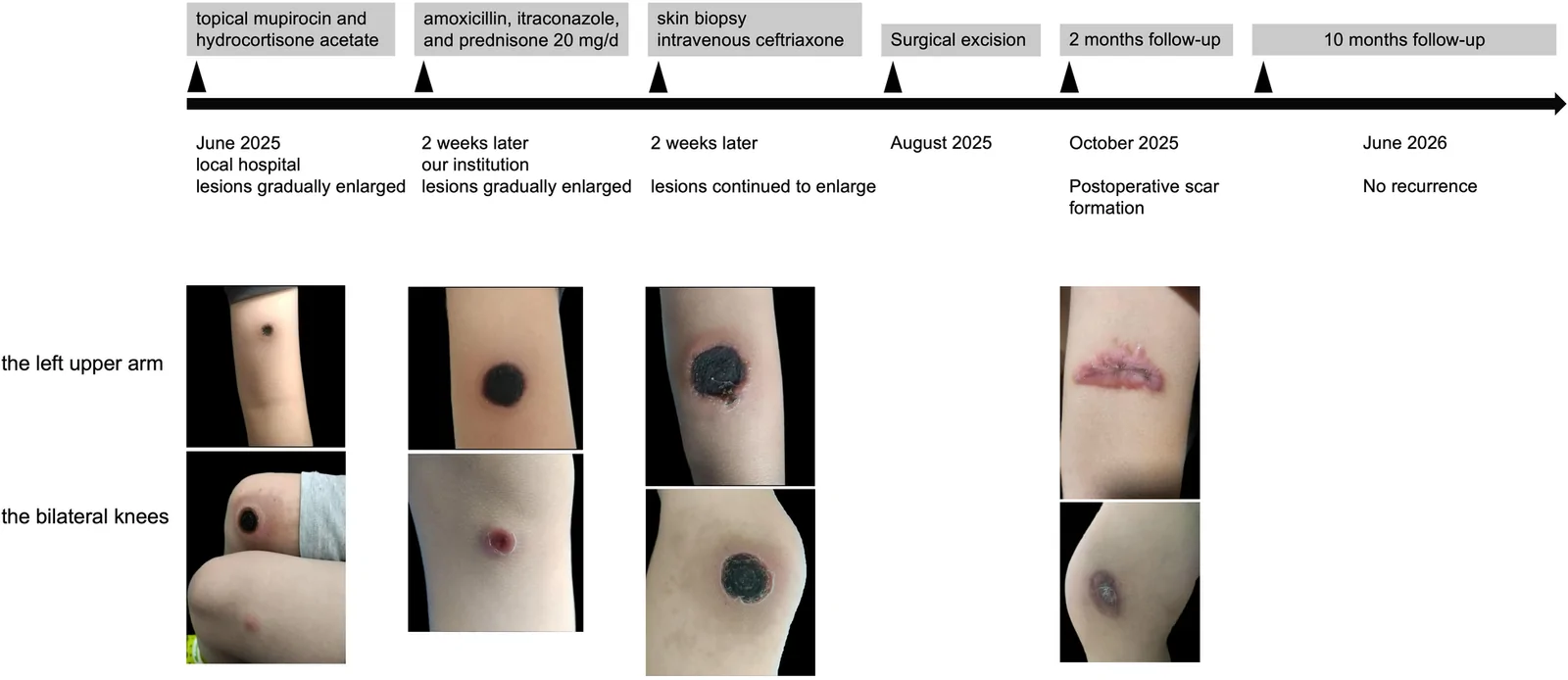
Timeline of disease, intervention, and outcome.

**Figure 2:**
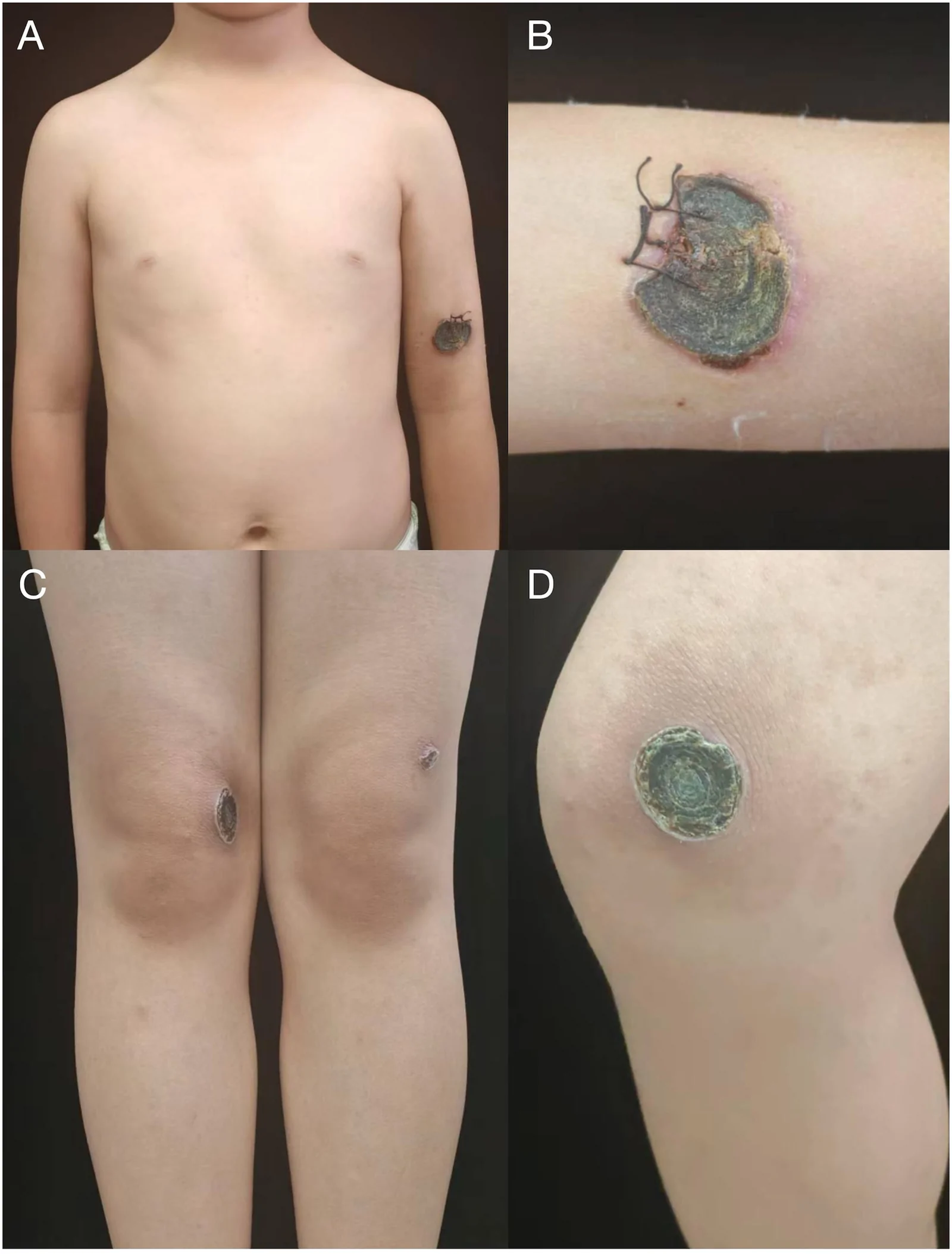
Skin examination. Eschar-like ulcers measuring 4 cm on the left upper arm **(A,B)**, 0.5 cm on the left knee and 3 cm on the right knee **(C)**, with a closer view of the right knee lesion in **(D).**

Laboratory investigations including routine blood tests, biochemical analyses, coagulation studies, urinalysis, stool evaluations, C-reactive protein, procalcitonin, Epstein–Barr virus DNA testing, TORCH (toxoplasmosis, other infections, rubella, cytomegalovirus, and herpes simplex virus) screening, and abdominal ultrasonography were unremarkable. Chest computed tomography showed mildly blurred bilateral lung markings and slightly decreased transparency in the lower lobes. Bacterial culture of skin secretions yielded *Kocuria* spp., *Corynebacterium* spp., and *Brachybacterium* spp. Metagenomic next-generation sequencing (mNGS) identified *Corynebacterium jeikeium.*

A skin biopsy from the left upper arm performed at our hospital revealed partial cutaneous ulceration and the remaining epidermis demonstrated mild acanthosis with multilocular intraepidermal vesiculation and focal basal cell liquefactive degeneration. A dense nodular lymphoid infiltrate involved the entire dermis and extended into the subcutaneous adipose tissue. A subset of large atypical lymphoid cells exhibited nuclear enlargement, marked hyperchromasia, irregular nuclear contours, and frequent mitotic figures. In the subcutis, there was fibrinoid necrosis of vessel walls and focal coagulative necrosis with abundant nuclear debris (Figure 3). Given the absence of systemic evidence of infection, the lack of clinical response to antimicrobial therapy, and the histopathological findings supporting a lymphoproliferative disorder rather than an infectious process, the organisms detected by culture and mNGS were considered to represent secondary colonization of the ulcerated lesions rather than causative infection.

**Figure 3:**
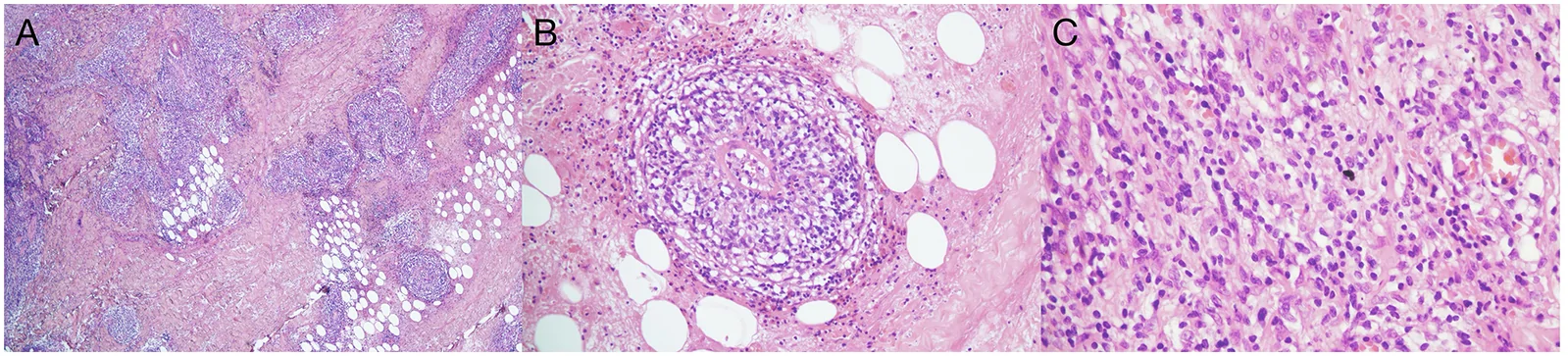
Histopathologic findings. **(A)** Hematoxylin and eosin (HE) staining showing a nodular infiltrate of atypical lymphoid cells involving the dermis and subcutaneous tissue (×40). **(B)** Prominent infiltration and destruction of vessel walls by atypical lymphoid cells, accompanied by fibrinoid necrosis of vascular walls and surrounding tissue necrosis (×200). **(C)** Dense infiltration of medium-to-large atypical lymphoid cells with irregular nuclear contours and frequent mitotic figures (×400).

Immunohistochemical staining revealed that the neoplastic cells were positive for CD2 and CD5, with partial expression of CD3, focal expression of CD7, and predominant expression of CD4. CD8 showed partial expression, whereas CD30 was strongly and diffusely expressed in more than 75% of neoplastic cells. The tumor cells were negative for CD20, CD56, TCR*δ*,ALK, and EMA, while *β*F1 was positive. TIA-1 was partially expressed, and granzyme B was expressed in a small subset of cells. CD31 staining highlighted only scattered residual endothelial cells without a continuous endothelial lining in vessels infiltrated and disrupted by neoplastic cells, consistent with vascular wall destruction. The Ki-67 proliferation index was approximately 50% (Figure 4). Epstein–Barr virus-encoded RNA (EBER1/2) *in situ* hybridization was negative. A clonal T-cell receptor gamma gene rearrangement was detected.

**Figure 4:**
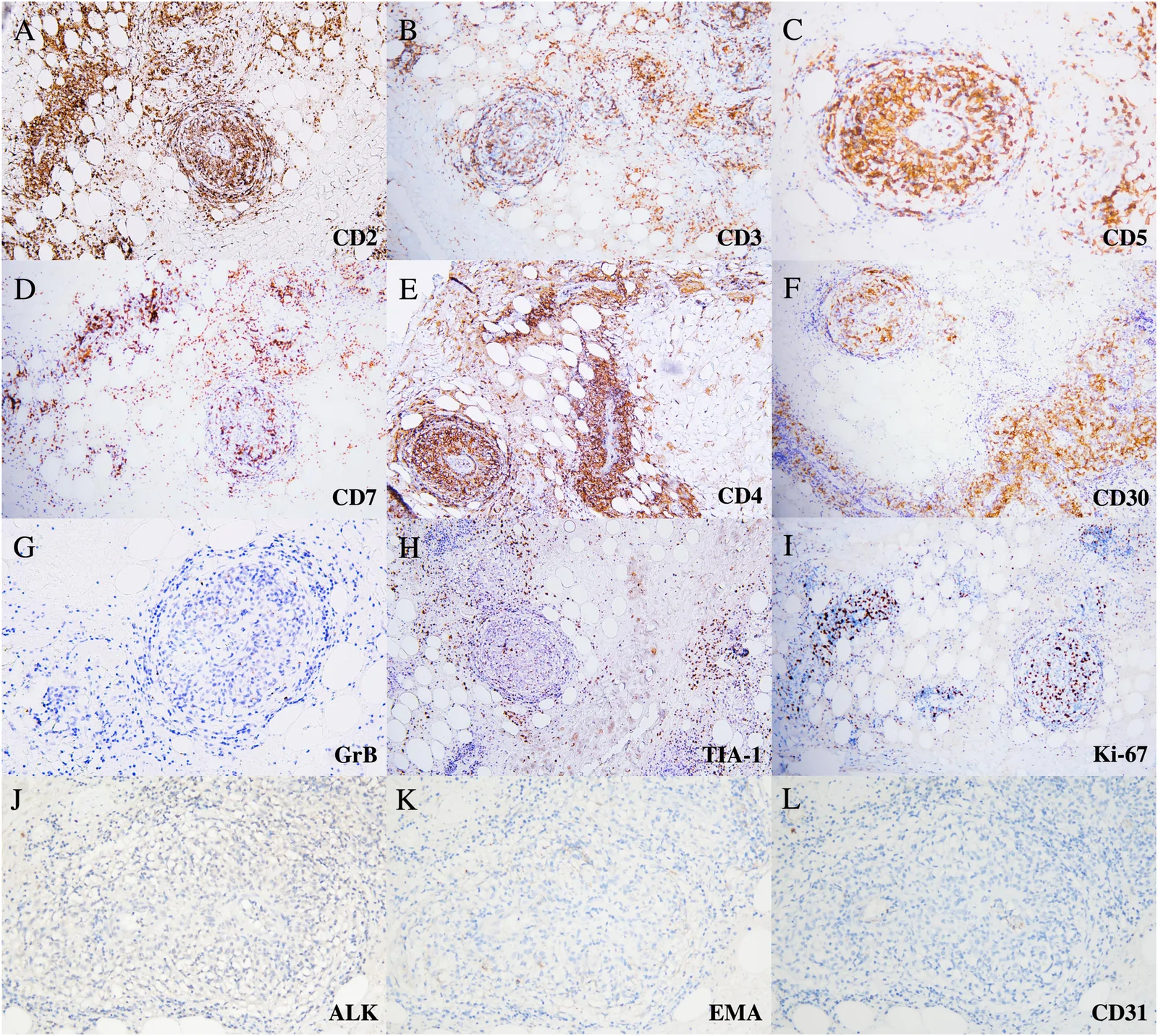
Immunohistochemical findings. **(A)** CD2 expression in neoplastic cells. **(B)** Partial expression of CD3. **(C)** CD5 expression in neoplastic cells. **(D)** Focal expression of CD7. **(E)** Predominant expression of CD4. **(F)** Strong and diffuse expression of CD30 in more than 75% of neoplastic cells. **(G)** Granzyme B expression in a small subset of neoplastic cells. **(H)** Partial expression of TIA-1. **(I)** Ki-67 proliferation index of approximately 50%. **(J)** Negative ALK expression. **(K)** Negative EMA expression. **(L)** CD31 immunostaining highlighted only scattered residual endothelial cells in vessels infiltrated and disrupted by neoplastic cells, without a continuous endothelial lining. Original magnification: A, B, D-F, H, I, ×100; C, G, J-L, ×200.

Taken together, the absence of systemic symptoms, lymphadenopathy, or extracutaneous involvement on imaging, together with the skin-limited presentation and negative ALK and EMA expression, supported the diagnosis of C-ALCL rather than systemic ALCL with secondary cutaneous involvement. Surgical excision was performed. No recurrence was observed during the 10-month follow-up period.

## Discussion

Primary cutaneous CD30+ lymphoproliferative disorders (LPDs) represent a spectrum of diseases and constitute the second most common group of cutaneous T-cell lymphomas after mycosis fungoides (2, 5, 6). This disease spectrum encompasses primary cutaneous anaplastic large cell lymphoma (C-ALCL) on one end and lymphomatoid papulosis (LyP) on the other, with borderline lesions exhibiting intermediate clinical and pathological features (6). It is most commonly seen in elderly patients and is uncommon in children (1, 2). C-ALCL presents as solitary or localized reddish-brown nodules or tumors (>2 cm), with rapid growth and common ulceration. It typically presents as either a solitary lesion or clustered lesions limited to one anatomical site, commonly located in the head, neck, and extremities, although multifocal disease occurs in 20% of cases (7, 8). Spontaneous partial or complete regression occurs uncommonly in C-ALCL (10% to 42%), and relapse is common (9). Our patient was an 8-year-old boy who developed isolated erythematous nodules at multiple anatomical sites, including the left upper arm and bilateral knees. The lesions ulcerated and formed black eschar-like ulcers with the largest measuring 4 cm × 4 cm, and showed no spontaneous regression.

The histopathologic features of C-ALCL are variable and nonspecific, typically showing a diffuse dermal infiltrate of medium to large anaplastic, pleomorphic, immunoblastic cells with abundant cytoplasm. “Hallmark” cells with eccentric “horseshoe” or “kidney-shaped” nuclei may be observed in C-ALCL. By definition, more than 75% of the tumor cells must express CD30 (2, 9). Histopathological examination of our patient revealed a diffuse dermal infiltrate of CD30+ large anaplastic cells with an angiocentric and angiodestructive pattern. Among previously published individual cases of C-ALCL with prominent angiocentric/angiodestructive features, the youngest patient was a 21-year-old man reported by Fuertes et al. (10). To the best of our knowledge, our 8-year-old patient therefore represents the youngest reported case of C-ALCL with this distinctive histopathological pattern. Our findings expand the clinicopathological spectrum of C-ALCL by demonstrating that, although this entity predominantly affects older adults, it can also occur in pediatric patients and may exhibit a distinctive angiotropic growth pattern.

The diagnosis of our case was challenging, yet accurate diagnosis is of paramount importance as it directly determines therapeutic decisions and prognostic assessment. Our case exhibited angiotropic histopathological features, necessitating differentiation from LyP type E as well as aggressive lymphomas characterized by an angioinvasive growth pattern. LyP is clinically characterized by a variable number of recurrent, self-healing papulonodular lesions (typically <2 cm), often manifesting as eschar-like ulcerations. Unlike C-ALCL, which typically presents as a solitary persistent lesion, lymphomatoid papulosis exhibits a characteristic waxing-and-waning course, with new lesions emerging and others regressing spontaneously within weeks (2, 9). In 2013, Kempf et al. (11) described a unique form of LyP characterized by hemorrhagic eschar-like ulcers clinically and angioinvasion histologically, proposed a designation LyP type E. Our patient presented with characteristic eschar-like ulcers on both lower extremities and the left upper extremity, measuring 0.5 cm, 3 cm, and 4 cm in diameter, respectively. In contrast to LyP type E, the lesions were localized, larger in size, and showed no spontaneous regression, favoring a clinical diagnosis of C-ALCL over LyP. This C-ALCL variant with prominent angiocentric/angiodestructive histopathological features was first described by Kempf et al. (12), with additional cases subsequently reported by other investigators (4, 10, 13). As this C-ALCL variant and LyP type E are histopathologically indistinguishable (12), clinical parameters such as lesion size, solitary or multifocal presentation, and the presence or absence of spontaneous regression during the disease course are of great significance in the differential diagnosis between these two entities. Considering that some patients with LyP type E may present with a solitary persistent lesion at a certain point during the disease course, distinction between these two entities can sometimes only be achieved during follow-up (12). An initial four to eight-week period of observation for all patients with primary cutaneous CD30+ lymphomas may be recommended, as spontaneous regression is possible and a definite diagnosis can normally be made (10, 14). Kempf et al. suggested that it cannot be entirely excluded that angiocentric C-ALCL may precede angiocentric LyP in those patients, which further reiterates the concept that LyP and C-ALCL comprise a clinicopathologic spectrum (12).

In addition to LyP type E, aggressive lymphomas characterized by an angioinvasive growth pattern must also be excluded, because their prognosis and management differ markedly from those of C-ALCL. Extranodal NK/T-cell lymphoma (ENKTL) is a non-Hodgkin lymphoma occurring at extranodal sites, originating from NK cells or T cells, with frequent dissemination to extracutaneous sites and an unfavorable prognosis. This disease is closely associated with Epstein–Barr virus (EBV) infection. The neoplastic cells typically express cytoplasmic CD3*ε*, CD56, and cytotoxic molecules. EBER positivity is a characteristic and diagnostically important feature of ENKTL. In our patient, EBER1/2 *in situ* hybridization was negative and the neoplastic cells lacked CD56 expression, strongly arguing against this diagnosis (11). Primary cutaneous *γ*/*δ* T-cell lymphoma (pcGDTCL) is a highly aggressive cutaneous T-cell lymphoma characterized by clonal proliferation of malignant *γ*/*δ* T cells. The majority of patients present with B symptoms, and some develop hemophagocytic syndrome, resulting in poor prognosis. By definition, pcGDTCL must express TCR*δ* (*γ*/*δ* chain of TCR) and lack the expression of *β*F1 (*α*/*β* chain of TCR). It is typically CD56+ with coexpression of cytotoxic markers (e.g., TIA-1, granzyme B), whereas atypical lymphoid cells in C-ALCL usually express *β*F1 and are negative for TCR*δ* (9, 11). Distinguishing this C-ALCL variant from these aggressive lymphomas is of paramount importance, as misinterpretation of the angiocentric and angiodestructive growth pattern may result in unnecessary aggressive therapy and inaccurate prognostic assessment.

C-ALCL is considered an indolent lymphoma, and the prognosis is usually favorable with a 10-year disease-related survival exceeding 90% (7). Recent studies have demonstrated that lesions larger than 5 cm and increased serum lactate dehydrogenase were associated with a poor prognosis in C-ALCL, whereas the survival of patients with C-ALCL was unaffected by the anatomical locations of skin lesions, localized or multifocal involvement and other pathological factors (15). Due to the limited number of reported cases, no systematic studies have been conducted to investigate the prognostic factors associated with this C-ALCL variant. Despite the worrisome histological features, all these patients remained well and disease-free after years of follow-up, with no progression to systemic lymphoma observed (12).

The management of C-ALCL should be tailored according to the extent and severity of skin involvement. For solitary or localized lesions of C-ALCL, the consensus first-line therapies are local approaches such as surgical excision or radiotherapy. These interventions are highly effective, achieving complete remission in approximately 95% of patients with localized disease (16). For multifocal C-ALCL confined to the skin, low-dose methotrexate and other systemic or skin-directed approaches may be selected according to the number, distribution, and burden of the lesions. Multiagent chemotherapy is generally reserved for patients with extracutaneous dissemination rather than those with skin-limited multifocal disease (16). Brentuximab vedotin may be considered for previously treated C-ALCL requiring systemic therapy (8). However, the best therapeutic strategy for multifocal C-ALCL remains to be identified. So far, no pediatric-specific consensus treatment guidelines are currently available. Surface brachytherapy has been reported for pediatric C-ALCL, with one study describing complete resolution of lesions in a 6-year-old girl following treatment, with no recurrence observed during 21 months of follow-up (17). Given the specific risks associated with radiotherapy in this age group, as well as concerns from both clinicians and parents regarding systemic agents, surgical excision was ultimately selected for this child despite multiple ulcerated lesions on the extremities, as the disease remained localized, even with the risk of residual scarring. No recurrence has been observed during 10-month follow-up. Unfortunately, the boy developed postoperative scarring, with partial involvement at joint sites, which may compromise long-term quality of life. Therefore, early scar prevention interventions following surgery are recommended to improve long-term outcomes.

We report the youngest patient to date with C-ALCL characterized by prominent angiotropism as the histopathological feature. This case highlights the diagnostic challenges posed by this rare histological variant and underscores the importance of early and accurate diagnosis to avoid overtreatment while enabling appropriate prognostic assessment. Our findings expand the current clinicopathological spectrum of C-ALCL and provide a practical diagnostic framework for the evaluation of similar cases in the future. Nevertheless, complete diagnostic distinction may not always be possible at the initial presentation, emphasizing the importance of long-term clinical follow-up to establish the diagnosis and monitor disease progression.

## Patient perspective

Because of safety concerns, the patient's parents opted for surgical treatment. Although postoperative scarring developed near the joints, the boy has not experienced any restriction of joint movement. He considered the postoperative scars far more acceptable than the previous large, black eschar-like ulcers. The boy and his parents were highly satisfied with the treatment outcome and indicated that they may consider scar treatment in the future for cosmetic reasons.

### Limitations

Several limitations should be acknowledged. First, as a single case, it cannot establish the prognostic significance of angioinvasive features in pediatric C-ALCL. Second, the follow-up period of 10 months was relatively short, and longer follow-up is needed to assess the long-term clinical course and risk of recurrence. In addition, molecular and cytogenetic studies, including assessment of DUSP22 and TP63 rearrangements, were not performed. Although these tests are not required for the diagnosis of C-ALCL, they could provide additional insight into the molecular characteristics of this unusual variant.

## Data Availability

The original contributions presented in the study are included in the article/Supplementary Material, further inquiries can be directed to the corresponding author.
